# Open Emergent Groin Hernia Repair: Anterior or Posterior Approach?

**DOI:** 10.3389/jaws.2022.10586

**Published:** 2022-07-21

**Authors:** V. Rodrigues-Gonçalves, M. Verdaguer, M. Moratal, R. Blanco, A. Bravo-Salva, J. A. Pereira-Rodíguez, M. López-Cano

**Affiliations:** ^1^ Abdominal Wall Surgery Unit, General Surgery Department, Hospital Universitari Vall d'Hebron, Universitat Autónoma de Barcelona, Barcelona, Spain; ^2^ General Surgery, Hospital del Mar, Parc de Salut Mar, Barcelona, Spain; ^3^ Department de Ciéncies Experimentals i de la Salut, Universitat Pompeu Fabra, Barcelona, Spain

**Keywords:** open preperitoneal hernia repair, incarcerated, strangulated, prosthetic mesh repair, emergent groin hernia

## Abstract

**Introduction:** The current literature has not yet provided a definitive conclusion on the best emergency groin hernia repair. The aim of this study was first to compare the short and long-term outcomes between open preperitoneal and anterior approach in emergency groin hernia repair and second to identify risk factors for postoperative complications, mortality, and recurrence.

**Materials and Methods:** This retrospective cohort study included patients who underwent emergency groin hernia repair between January 2010 and December 2018. Short and long-term outcomes were analyzed comparing approach and repair techniques. The predictors of complications and mortality were investigated using multivariate logistic regression. Cox regression multivariate analysis were used to explore risk factors of recurrence.

**Results:** A total of 316 patients met the inclusion criteria. The most widely used surgical techniques were open preperitoneal mesh repair (34%) and mesh plug (34%), followed by Lichtenstein (19%), plug and patch (7%) and tissue repair (6%). Open preperitoneal mesh repair was associated with lower rates of recurrence (*p* = 0.02) and associated laparotomies (*p* < 0.001). Complication and 90-day mortality rate was similar between the techniques. Multivariable analysis identified patients aged 75 years or older (OR, 2.08; 95% CI, 1.14–3.80; *p* = 0.016) and preoperative bowel obstruction (OR, 2.11; 95% CI, 1.20–3.70; *p* = 0.010) as risk factors for complications and Comprehensive Complication Index ≥26.2 as risk factor for 90-day mortality (OR, 44.76; 95% CI, 4.51–444.59; *p* = 0.01). Female gender was the only risk factor for recurrence.

**Conclusion:** Open preperitoneal mesh repair may be superior to other techniques in the emergency setting, because it can avoid the morbidity of associated laparotomies, with a lower long-term recurrence rate.

## Introduction

Nowadays the optimal surgical technique in emergency groin hernia repair remains controversial [1]. Open anterior, open posterior (preperitoneal) and laparoscopic approach with mesh in selected patients has been used safely and effectively [2–5]. However, the evidence is limited, and the choice of a particular approach seems to be based on the criteria and experience of the surgeon in charge [1]. A low-quality randomized study has reported benefits of the open preperitoneal approach in terms of lower incidence of second incisions compared to open anterior approach (Lichtenstein technique) [6]. Nevertheless, there is very scarce data evaluating the short and long-term results of the preperitoneal access in the emergency setting and the potential benefits of this technique remains unknown [6–8].

The primary aim of this study was to compare the short and long-term outcomes between open preperitoneal and open anterior approach in emergency groin hernia repair. Secondarily to identify risk factors for postoperative complications, mortality, and recurrence.

## Materials and Methods

### Patients and Definitions

This is a retrospective single-center cohort study of all adult patients who underwent emergency groin hernia repair for incarceration or strangulation at Vall d´Hebron University Hospital between January 2010 and December 2018, who were identified from a prospectively maintained database of the Abdominal Wall Surgery Unit of the Surgery Department of our hospital. Emergency groin hernia repair was defined as inguinal or femoral hernia repaired on an emergency basis as a consequence of acute incarceration or strangulation. Incarceration was defined as the inability to reduce the hernia mass into the abdomen and strangulation was defined by the evidence of compromised blood supply to herniated tissues according to the International Guidelines for the management of groin hernia [1]. Patients under 18 years and those who underwent elective surgery after manual or spontaneous reduction of the hernia content were excluded. The data was completed through a retrospective review from medical and surgical records. Data collected included: demographic and clinical information, operative details, short and long-term outcomes measures.

### Demographic and Clinical Information

Age, gender, body mass index (BMI), American Society of Anesthesiologists (ASA) class, Charlson score [9], cardiovascular disease, chronic obstructive pulmonary disease, chronic nephropathy, liver cirrhosis, ascites, neurocognitive disorders, diabetes, inmunosupression and smoking status were collected. Clinical and radiological evidence of preoperative bowel obstruction and duration of incarceration were included. The duration of incarceration was defined as the time elapsed from the start of incarceration referred by the patient until was admitted in the emergency area. Hernia variables included hernia side, type (indirect, direct, femoral, “pantaloon” and sliding) and hernia content.

### Operative Information

The surgical approach was classified as open anterior or open preperitoneal. An open transinguinal repair without entering the preperitoneal space using a tissue or mesh technique were considered an anterior approach. An open posterior access of the preperitoneal space without entering the inguinal canal anteriorly and with enough exposure of the Bogros space [10] to allow hernia repair with or without placement of a prosthesis were considered a preperitoneal approach. Repair techniques were categorized as: Lichtenstein, plug and patch, mesh plug, tissue repair and open preperitoneal mesh repair.

In cases when the surgeon´s choice was to perform an open preperitoneal approach, a transverse abdominal incision (8–10 cm in length) was made about two fingerbreadths above the symphysis pubis and two fingers outside the midline was performed. The dissection was carried successively through the skin, subcutaneous tissue, anterior rectus sheath and the oblique muscles aponeurosis (the transverse fascial incision was made the same length as the skin incision). The rectus muscle was retracted medially, and the transversalis fascia was incised to expose the hernia sac. The inferior epigastric vessels were divided as needed. The peritoneum was opened, and hernia contents were delivered, inspected, and reduced. In cases where an intestinal resection and anastomosis was required, it was performed through same incision. The peritoneum was closed after dissection of the vas and vessels off the hernia sac. By retracting the pelvis peritoneum and preperitoneal fat away from the posterior inguinal wall, direct and indirect as well as femoral hernias were recognized. The next step was the placement of the prosthesis. A mesh of polypropylene with minimum size 15 × 15 cms was used to completely cover and overlap the myopectineal orifice. The mesh was anchored, using one stitch of 2-0 synthetic absorbable monofilament to the Cooper´s ligament. A slit was made in the lateral border of the mesh to accommodate the spermatic cord. After spread of mesh prosthesis, layers were closed anatomically.

Following the definitions described above, the patients were grouped according to repair approach in open anterior and open posterior, and according to repair techniques in tissue repair, Lichtenstein, plug and patch, mesh plug, and open preperitoneal mesh repair. The different characteristics of the patients were compared first, between open anterior and open posterior groups, and second, between repair techniques groups.

Other operative details were collected: tissue or mesh repair, bowel resection, anesthesia type, intraoperative complications defined as visceral (i.e., intestinal), vascular (i.e., deep epigastric vessels or femoral vessels) and/or urinary bladder injuries. Midline laparotomy if needed was also collected. Type of surgical wounds were defined according to CDC classification [11]. Clean-contaminated wounds were defined as those in which the alimentary, genital, or urinary tract were entered under controlled conditions and without unusual contamination. Contaminated wounds were those in which there were major interruptions in sterile technique or significant spills from the gastrointestinal tract and incisions in which acute non-purulent inflammations were found.

Broad spectrum antibiotic are given systematically in emergency groin hernia repair and nasogastric tube decompression in cases of bowel obstruction. Anesthesia type was decided by the anesthesiologist. Surgical approach and repair technique were the surgeon´s choice. In cases of anterior approach with bowel resection needs (ischemic) the resection was done via inguinal incision or doing a midline infraumbilical laparotomy incision. In cases of open preperitoneal access the resection was done through same incision.

### Outcomes Definition and Follow-Up

Short- and long-term outcomes were compared according to the types of approach and repair techniques.

Short-term outcomes (within postoperative 90 days) evaluated were: length of hospital stay in days (admission-discharge), reoperations rate (not related to recurrences), mortality within 90 days of surgery and postoperative complications. Postoperative complication was defined as any condition that could prolong the length hospital stay or impact the outcomes. Complications were categorized according Clavien-Dindo grading system [12] and was measure using the Comprehensive Complication Index (CCI^®^, University of Zurich, Zurich, Switzerland) [13].

Long-term outcomes (after postoperative 90 days) evaluated were: recurrence and chronic postoperative inguinal pain. Recurrence was considered after physical examination by the surgeon, review of operative notes reporting repairs of recurrent ipsilateral hernia, or by telephone interviews with the patient using the Ventral Hernia Recurrence Inventory (VHRI) [14]. VHRI is a patient reported outcomes tool, which is considered an accurate method for evaluating recurrence of ventral hernia [15] and validated for inguinal hernia [14]. Chronic postoperative inguinal pain was defined as pain persisting continuously or intermittently for more than 3 months after surgery [16]. Chronic postoperative inguinal pain was assessed by telephone interview using the last question of the VHRI questionnaire: “Do you have pain or other physical symptoms at the site?”. Any positive responses to VHRI prompted a follow-up request for a physical exam. For those patients who did not respond to the follow up telephone interview or call, the last postoperative face-to-face visit was considered as the last follow-up date.

Routinely a follow-up visit was made 2 weeks after hospital discharge and depending on the presence of postoperative complications, more face-to-face visits were scheduled. To assess the presence of recurrence and chronic postoperative pain, telephone interviews were conducted at the time of this study.

Further analysis were performed to determine risk factors for postoperative complications, 90-day mortality and recurrence.

### Statistical Analysis

Continuous variables were presented as median and interquartile ranges (IQRs) and compared by using the Mann-Whitney U test. Categorical variables were presented as counts and percentages and compared by Chi-square test of Fisher´s exact test, when indicated. Two logistic regression models were built, one using postoperative complications as the outcome, and other using 90-day mortality. Cox regression multivariate analysis were used to explore risk factors of recurrence. Covariates included in the models were based on clinical consensus and according to significance in the univariate analysis (*p* < 0.1). The results of complications and 90-day mortality are presented as odds ratios with 95% confidence intervals and recurrence are presented as hazard ratio with 95% confidence intervals. Cumulative recurrence rate was estimated by the Kaplan-Meier method and tested for significance with the log-rank test. A value of *p* < 0.05 was considered significant. SPSS (IBMS SPSS Statistics 23) was used for statistical analysis.

## Results

### Patients

A total of 316 patients underwent emergency groin hernia repair at our institution were included. All the operations were performed through an open approach, of which 206 (65.2%) underwent an anterior approach and 110 (34.8%) an open preperitoneal approach. Mesh repair was performed in 296 patients (93.67%) and 20 patients (6.33%) underwent tissue repair (3 patients following preperitoneal approach and 17 anterior approach). The repair techniques used were Lichtenstein in 61 (19.3%) patients, plug and patch in 21 (6.6%), mesh plug in 107 (33.9%), preperitoneal mesh in 107 (33.9%) and tissue repair in 20 (6.3%) patients. The tissue repair techniques used were Bassini-Kirschner in 9 patients, Bassini in 4, Lotheissen-McVay in 4, Nyhus in 2, and Shouldice in 1 patient. In our series there were no bilateral hernia repairs. The characteristics of patients regarding type of approach are shown in Table 1 and regarding type of technique in Table 2.

**TABLE 1:** Patient Characteristics of Study Population according to the repair approach.

Variables	Total (*n* = 316)	Anterior approach (*n* = 206)	Preperitoneal approach (*n* = 110)	*p* Value
Age (yr)[median (IQR)]	78 (69–85)	77.5 (69–84)	80 (70–87)	0.085
Gender [n, (%)]				0.782
Male	147 (46.52)	97 (47.09)	50 (45.45)	
Female	169 (53.48)	109 (52.91)	60 (54.55)	
BMI (kg/m^2^) [median (IQR)]	24.8 (22.3–27.6)	25.1 (23–28)	23.7 (21.6–26.4)	0.010
ASA score				0.582
I/II [n, (%)]	179 (56.60)	119 (57.77)	60 (54.55)	
III/IV [n, (%)]	137 (43.35)	87 (42.23)	50 (45.45)	
Charlson score [median (IQR)]	5 (4–6)	5 (4–6)	5 (4–6)	0.800
Previous abdominal surgery [n, (%)]	137 (43.35)	90 (43.69)	47 (42.73)	0.869
Comorbidity [n, (%)]	259 (81.96)	168 (81.55)	91 (82.73)	0.796
Cardiovascular disease [n, (%)]	223 (70.57)	142 (68.93)	81 (73.64)	0.382
Chronic obstructive pulmonary disease [n, (%)]	65 (20.57)	43 (20.87)	22 (20.00)	0.855
Chronic nephropathy [n, (%)]	37 (11.71)	21 (10.19)	16 (14.55)	0.252
Liver cirrhosis [n, (%)]	10 (3.16)	9 (4.37)	1 (0.91)	0.094
Ascites [n, (%)]	10 (3.16)	8 (3.88)	2 (1.82)	0.318
Neurocognitive disorders [n, (%)]	48 (15.19)	30 (14.56)	18 (16.36)	0.671
Diabetes [n, (%)]	38 (12.03)	26 (12.62)	12 (10.91)	0.656
Immunosuppression [n, (%)]	18 (5.70)	12 (5.83)	6 (5.45)	0.892
Active smoking [n, (%)]	26 (8.23)	15 (7.28)	11 (10)	0.402
Comorbidity more than one [n, (%)]	167 (52.85)	109 (52.91)	58 (52.73)	0.975
Hernia type [n, (%)]				0.757
Inguinal indirect	76 (24.05)	46 (22.33)	30 (27.27)	
Inguinal direct	47 (14.87)	32 (15.53)	15 (13.64)	
Femoral	179 (56.65)	118 (57.28)	61 (55.45)	
Others	14 (4.43)	10 (4.85)	4 (3.64)	
Hernia side [n, (%)]				0.210
Right	189 (59.81)	118 (57.28)	71 (64.55)	
Left	127 (40.19)	88 (42.72)	39 (35.45)	
Recurrent hernia [n, (%)]	56 (17.72)	36 (17.48)	20 (18.18)	0.876
Hernia sac contents [n, (%)]				0.194
Omentum	45 (14.24)	31 (15.05)	14 (12.73)	
Small bowel	194 (61.39)	124 (60.19)	70 (63.64)	
Colon	23 (7.28)	13 (6.31)	10 (9.09)	
Bladder	3 (0.95)	1 (0.49)	2 (1.82)	
Appendix	4 (1.27)	2 (0.97)	2 (1.82)	
Other	18 (5.70)	10 (4.85)	8 (7.27)	
Not reported	8 (2.53)	6 (2.91)	2 (1.82)	
Reported as empty at the moment of opening	21 (6.65)	19 (9.22)	2 (1.82)	
Necrotic contents [n, (%)]	81 (25.63)	46 (22.33)	35 (31.82)	0.066
Preoperative bowel obstruction [n, (%)]	165 (52.22)	101 (49.03)	64 (58.18)	0.121
Duration of incarceration [median (IQR)]	24 (11–72)	24 (10–72)	24.5 (12–72)	0.833
Grade of contamination [n, (%)]				0.975
Clean	235 (74.37)	154 (74.76)	81 (73.64)	
Clean/contaminated	64 (20.25)	41 (19.90)	23 (20.91)	
Contaminated	17 (5.38)	11 (5.34)	6 (5.45)	
Bowel resection performed [n, (%)]	66 (20.89)	38 (18.45)	28 (25.45)	0.144
Type of anesthesia [n, (%)]				0.006
Spinal	148 (46.84)	110 (53.40)	38 (34.55)	
Local alone	7 (2.22)	4 (1.94)	3 (2.73)	
General	161 (50.95)	92 (44.66)	69 (62.73)	
Required midline laparotomy [n, (%)]	24 (7.59)	19 (9.22)	5 (4.55)	0.135
Intraoperative complications [n, (%)]	17 (5.38)	10 (4.85)	7 (6.36)	0.571
Postoperative complications [n (%)]	152 (48.1)	99 (48.06)	53 (48.18)	0.983
Comprehensive complication index [median (IQR)]	8.7 (0–29.6)	8.7 (0–29.6)	8.7 (0–29.6)	0.856
Clavien Dindo classification of postoperative complications [n (%)]				0.971
None	178 (56.33)	115 (55.83)	63 (57.27)	
I/II	91 (28.80)	59 (28.64)	32 (29.09)	
III/IV	26 (8.23)	18 (8.74)	8 (7.27)	
V	21 (6.65)	14 (6.80)	7 (6.36)	
Reoperation [n, (%)]	13 (4.11)	9 (4.37)	4 (3.64)	0.755
Length of stay (days) [median (IQR)]	4 (2–7.5)	4 (2–7)	4 (2–8)	0.391
Recurrence [n, (%)]	27 (8.5)	23 (7.3)	4 (1.3)	0.023
Chronic postoperative inguinal pain [n, (%)]	7 (2.2)	5 (2.4)	2 (1.8)	0.818

**TABLE 2:** Patient Characteristics of Study Population according to the repair technique.

Variables	Total (*n* = 316)	Lichtenstein (*n* = 61)	Plug and patch (*n* = 21)	Mesh plug (*n* = 107)	Tissue repair (*n* = 20)	Preperitoneal mesh (*n* = 107)	*p* Values
Age (yr)[median (IQR)]	78 (69–85)	74 (67–83)	78 (73–81)	78 (70–84)	80.5 (71–86)	81 (70–87)	0.188
Gender [n, (%)]							< 0.001
Male	147 (46.52)	43 (70.49)	13 (61.90)	36 (33.64)	7 (35.00)	48 (44.86)	
Female	169 (53.48)	18 (29.51)	8 (38.10)	71 (66.36)	13 (65.00)	59 (55.14)	
BMI (kg/m^2^) [median (IQR)]	24.8 (22.3–27.6)	26.6 (24.3–29)	25.1 (22.7–29.3)	24.9 (22.3–27.2)	23.35 (22.3–27.1)	23.85 (21.75–26.4)	0.004
ASA score							0.843
I/II [n, (%)]	179 (56.65)	38 (62.30)	13 (61.90)	59 (55.14)	11 (55.00)	58 (54.21)	
III/IV [n, (%)]	137 (43.35)	23 (37.70)	8 (38.10)	48 (44.86)	9 (45.00)	49 (45.79)	
Charlson score [median (IQR)]	5 (4–6)	5 (4–7)	6 (4–8)	5 (4–6)	4 (4–6)	5 (4–6)	0.417
Previous abdominal surgery [n, (%)]	137 (43.35)	26 (42.62)	12 (57.14)	49 (45.79)	5 (25.00)	45 (42.06)	0.318
Comorbidity [n, (%)]	259 (81.96)	52 (85.25)	19 (90.48)	84 (78.5)	14 (70.00)	90 (84.11)	0.330
Cardiovascular disease [n, (%)]	223 (70.57)	44 (72.13)	17 (80.95)	70 (65.42)	12 (60.00)	80 (74.77)	0.341
Chronic obstructive pulmonary disease [n, (%)]	65 (20.57)	13 (21.31)	5 (23.81)	22 (20.56)	3 (15.00)	22 (20.56)	0.970
Chronic nephropathy [n, (%)]	37 (11.71)	6 (9.84)	3 (14.29)	8 (7.48)	4 (20.00)	16 (14.95)	0.329
Liver cirrhosis [n, (%)]	10 (3.16)	2 (3.28)	0 (0)	6 (5.61)	1 (5.00)	1 (0.93)	0.316
Ascites [n, (%)]	10 (3.16)	2 (3.28)	0 (0)	5 (4.67)	1 (5.00)	2 (1.87)	0.683
Neurocognitive disorders [n, (%)]	48 (15.19)	8 (13.11)	6 (28.57)	15 (14.02)	1 (5.00)	18 (16.82)	0.280
Diabetes [n, (%)]	38 (12.03)	12 (19.67)	2 (9.52)	12 (11.21)	0 (0)	12 (11.21)	0.174
Inmunosupression [n, (%)]	18 (5.7)	2 (3.28)	2 (9.52)	7 (6.54)	2 (10.00)	5 (4.67)	0.685
Active smoking [n, (%)]	26 (8.23)	4 (6.56)	4 (19.05)	7 (6.54)	1 (5.00)	10 (9.35)	0.362
Comorbidity more than one [n, (%)]	167 (52.85)	30 (49.18)	15 (71.43)	55 (51.4)	10 (50.00)	57 (53.27)	0.493
Hernia type [n, (%)]							< 0.001
Inguinal indirect	76 (24.05)	29 (47.54)	8 (38.10)	5 (4.67)	5 (25.00)	29 (27.10)	
Inguinal direct	47 (14.87)	18 (29.51)	5 (23.81)	7 (6.54)	3 (15.00)	14 (13.08)	
Femoral	179 (56.65)	8 (13.11)	6 (28.57)	94 (87.85)	11 (55.00)	60 (56.07)	
Others	14 (4.43)	6 (9.84)	2 (9.52)	1 (0.93)	1 (5.00)	4 (3.74)	
Hernia side [n, (%)]							0.029
Right	189 (59.81)	28 (45.90)	17 (80.95)	61 (57.01)	13 (65.00)	70 (65.42)	
Left	127 (40.19)	33 (54.10)	4 (19.05)	46 (42.99)	7 (35.00)	37 (34.58)	
Recurrent hernia [n, (%)]	56 (17.72)	12 (19.67)	4 (19.05)	16 (14.95)	4 (20.00)	20 (18.69)	0.926
Hernia sac contents [n, (%)]							0.031
Omentum	45 (14.24)	9 (14.75)	0 (0)	21 (19.63)	2 (10.00)	13 (12.15)	
Small bowel	194 (61.39)	29 (47.54)	19 (90.48)	66 (61.68)	10 (50.00)	70 (65.42)	
Colon	23 (7.28)	6 (9.84)	1 (4.76)	3 (2.80)	4 (20.00)	9 (8.41)	
Bladder	3 (0.95)	1 (1.64)	0 (0)	0 (0)	0 (0)	2 (1.87)	
Appendix	4 (1.27)	0 (0)	0 (0)	2 (1.87)	1 (5.00)	1 (0.93)	
Other	18 (5.70)	3 (4.92)	1 (4.76)	5 (4.67)	1 (5.00)	8 (7.48)	
Not reported	8 (2.53)	3 (4.92)	0 (0)	3 (2.80)	0 (0)	2 (1.87)	
Reported as empty at the moment of opening	21 (6.65)	10 (16.39)	0 (0)	7 (6.54)	2 (10.00)	2 (1.87)	
Necrotic contents [n, (%)]	81 (25.63)	7 (11.48)	1 (4.76)	31 (28.97)	9 (45.00)	33 (30.84)	0.002
Preoperative bowel obstruction [n, (%)]	165 (52.22)	25 (40.98)	12 (57.14)	53 (49.53)	12 (60.00)	63 (58.88)	0.200
Duration of incarceration [median (IQR)]	24 (11–72)	16 (11–48)	10 (6–48)	47 (12–72)	41 (18.5–96)	24 (12–72)	0.024
Grade of contamination [n, (%)]							0.050
Clean	235 (74.37)	51 (83.61)	18 (85.71)	75 (70.09)	11 (55.00)	80 (74.77)	
Clean/contaminated	64 (20.25)	8 (13.11)	3 (14.29)	27 (25.23)	5 (25.00)	21 (19.63)	
Contaminated	17 (5.38)	2 (3.28)	0 (0)	5 (4.67)	4 (20.00)	6 (5.61)	
Bowel resection performed [n, (%)]	66 (20.89)	6 (9.84)	1 (4.76)	26 (24.3)	8 (40.00)	25 (23.36)	0.010
Type of anesthesia [n, (%)]							0.016
Spinal	148 (46.84)	36 (59.02)	15 (71.43)	53 (49.53)	8 (40.00)	36 (33.64)	
Local alone	7 (2.22)	0 (0)	0 (0)	3 (2.80)	1 (5.00)	3 (2.80)	
General	161 (50.95)	25 (40.98)	6 (28.57)	51 (47.66)	11 (55.00)	68 (63.55)	
Required midline laparotomy [n, (%)]	24 (7.59)	3 (4.92)	2 (9.52)	8 (7.48)	7 (35.00)	4 (3.74)	< 0.001
Intraoperative complications [n, (%)]	17 (5.38)	5 (8.2)	1 (4.76)	3 (2.8)	1 (5.00)	7 (6.54)	0.618
Postoperative Complications [n (%)]	152 (48.1)	26 (42.62)	10 (47.62)	53 (49.53)	11 (55.00)	52 (48.6)	0.876
Comprehensive complication index [median (IQR)]	8.7 (0–29.6)	8.7 (0–29.6)	8.7 (0–30.8)	8.7 (0–26.2)	60.6 (19.25–100)	8.7 (0–29.6)	0.020
Clavien Dindo classification of postoperative complications [n (%)]							0.297
None	178 (56.33)	38 (62.30)	11 (52.38)	59 (55.14)	9 (45.00)	61 (57.01)	
I/II	91 (28.80)	14 (22.95)	7 (33.33)	34 (31.78)	4 (20.00)	32 (29.91)	
III/IV	26 (8.23)	6 (9.84)	2 (9.52)	8 (7.48)	2 (10.00)	8 (7.48)	
V	21 (6.65)	3 (4.92)	1 (4.76)	6 (5.61)	5 (25.00)	6 (5.61)	
Reoperation [n, (%)]	13 (4.11)	2 (3.28)	1 (4.76)	6 (5.61)	0 (0)	4 (3.74)	0.803
Length of stay (days) [median (IQR)]	4 (2–7.5)	3 (2–6)	4 (2–7)	4 (2–8)	4 (2–9)	4 (2–8)	0.891
Recurrence [n, (%)]	27 (8.5)	4 (6.6)	1 (4.8)	17 (15.9)	1 (5)	4 (3.7)	0.020
Chronic postoperative inguinal pain [n, (%)]	7 (2.2)	0 (0)	0 (0)	5 (4.6)	0 (0)	2 (1.8)	0.365

Patients with an anterior approach had a higher BMI and a majority of spinal anesthesia, while the open preperitoneal approach group had a lower BMI (*p* = 0.01) and more general anesthesia (*p* = 0.006). However, the clinical relevance is unlikely since the median differences are small. When performing comparisons by the different groups of repair techniques, again the differences can be seemingly meaningful, and the clinical relevance should be considered carefully. A greater number of female patients underwent mesh plug repairs, open preperitoneal and tissue repair, while the Lichtenstein and plug and patch techniques were used more in men (*p* < 0.001). Patients with higher BMI underwent more frequent Lichtenstein and plug and patch techniques (*p* = 0.004). Indirect hernias were mostly repaired with Lichtenstein and femoral hernias with mesh plug, open preperitoneal, and tissue repair (*p* < 0.001). In those patients with the longest incarceration duration, with necrotic contents and in whom an intestinal resection was performed, the most frequently performed techniques were the tissue repair, mesh plug, and open preperitoneal. In the Lichtenstein and plug and patch techniques, there was a greater use of spinal anesthesia with respect to tissue repair, mesh plug, and open preperitoneal where general anesthesia was the most widely used anesthetic technique (*p* = 0.016). Patients with tissue repair more frequently required the association of a midline laparotomy, while those who underwent an open preperitoneal approach were those who least needed it (*p* < 0.001).

### Postoperative Complications

The overall postoperative complications rate was 48.1% (152/316). There were no significant differences in morbidity between an anterior or open preperitoneal approach (*p* = 0.983), and between the different repair techniques (*p* = 0.876). There were no differences between the patients who underwent mesh repair and those with tissue repair (*p* = 0.523). Patients with major complications (Clavien-Dindo ≥3A) were 47 (14.8%), without significant differences regarding the type of approach (*p* = 0.971) or repair technique (*p* = 0.297). There were no differences regarding the CCI^®^ according to the type of approach (*p* = 0.856); however, by surgical techniques, tissue repair was associated with higher CCI^®^ compared to the other repair techniques (*p* = 0.02). Surgical reintervention was required by 13 patients for small bowel obstruction (n = 4), intestinal ischemia (*n* = 2), intra-abdominal abscess (*n* = 2), wound infection (*n* = 2), anastomotic leak (*n* = 1), intestinal perforation (*n* = 1) and wound hematoma (*n* = 1). The 90-day mortality was 8.5% (N = 27) and no statistically significant difference was seen between surgical approach (*p* = 0.799) or repair technique (*p* = 0.923) groups.

### Long-Term Outcomes According to Surgical Approach and Repair Techniques

**FIGURE 1:**
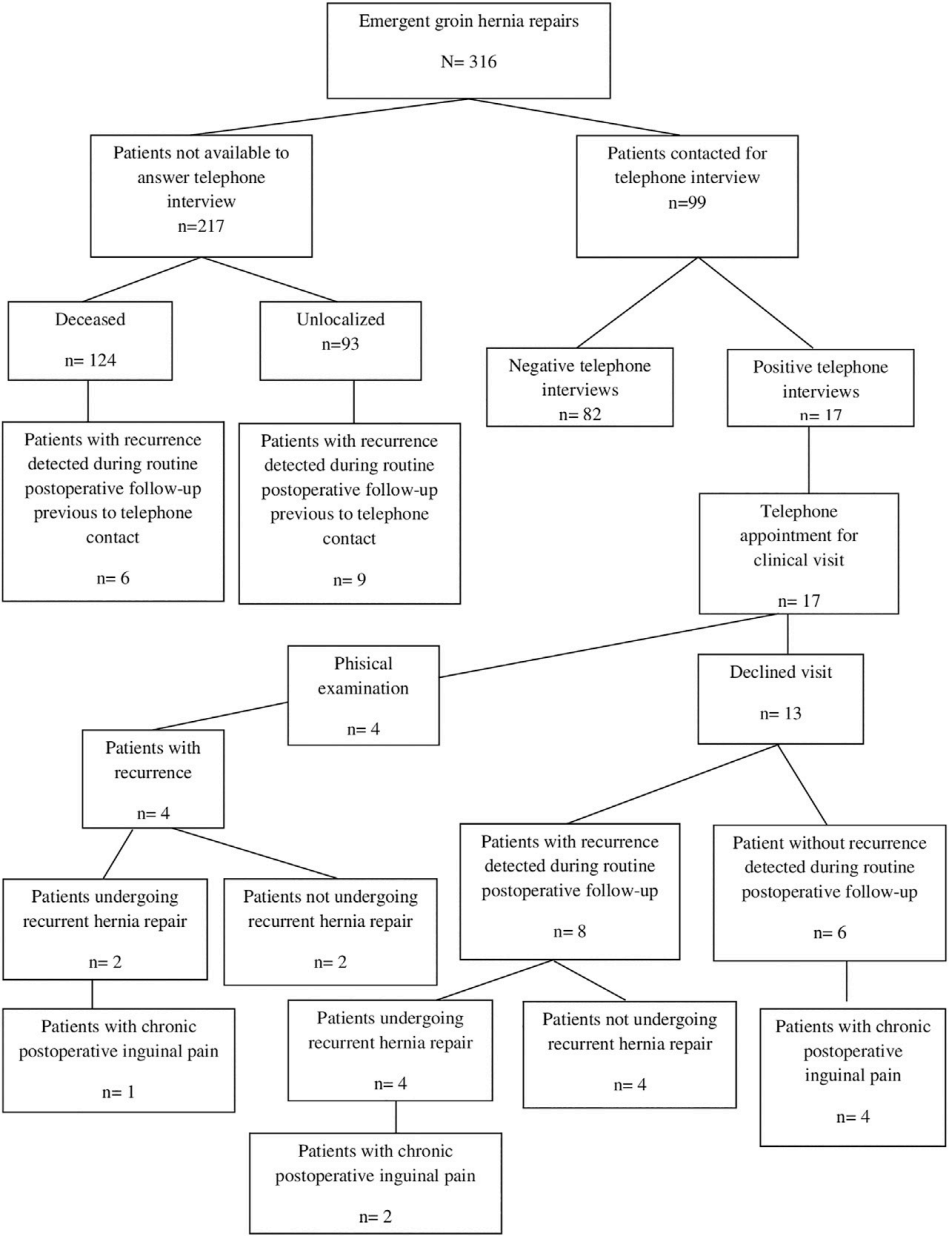
Flow-chart of study cohort and long-term outcomes.

The median follow-up period was 13.31 months (IQR: 0.86–52.93). The recurrence rate of the whole series was 8.5% (*n* = 27). A total of 20 (74.1%) recurrences appears after femoral hernia repair, 4 (14.8%) in indirect and 3 (11.1%) in direct hernias. There were no differences in recurrence rates between patients who underwent mesh repair and those with tissue repair (*p* = 1). Figure 1 shows the flow-chart of included patients and long-term outcomes.

**FIGURE 2:**
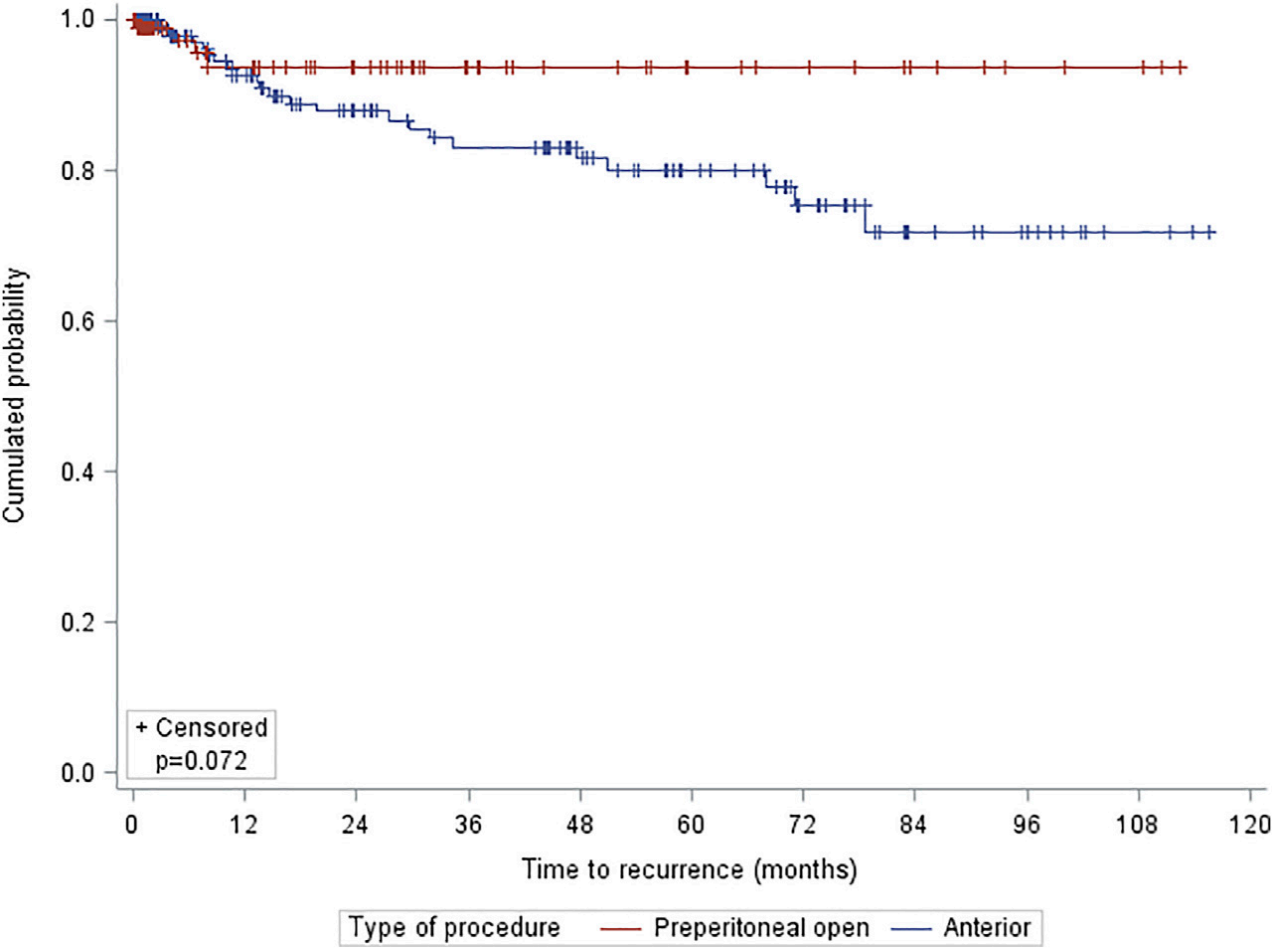
Kaplan-Meier estimates for long-term hernia recurrence by approach.

Patients with an open preperitoneal approach had a lower rate of recurrence compared with the anterior approach (*p* = 0.023). Regarding the cumulative recurrence, there were no differences according to the type of approach (*p* = 0.072, log rank) (Figure 2).

**FIGURE 3:**
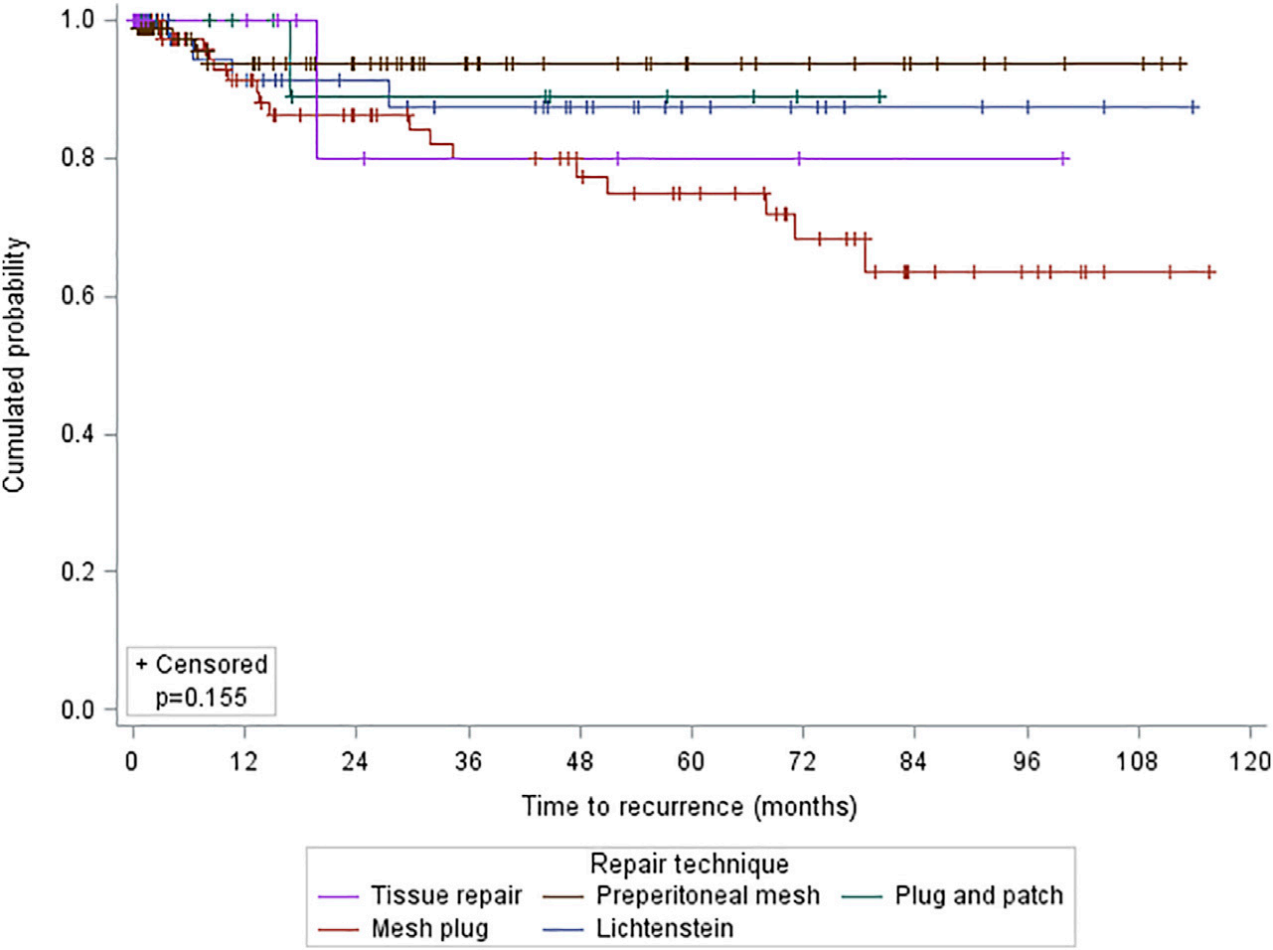
Kaplan-Meier estimates for long-term hernia recurrence by technique.

Higher recurrence rate was observed in patients with a mesh plug repair (*p* = 0.020). Regarding the cumulative recurrence, there were no differences according to the type of technique (*p* = 0.155, log rank) (Figure 3).

Concerning chronic postoperative inguinal pain, only 99 patients responded to the telephone interview and completed the VHRI questionnaire of them 7 presented chronic postoperative inguinal pain. No significant differences were found according to the type of approach (*p* = 0.818) or the type of surgical technique (*p* = 0.363).

### Risk Factors of Postoperative Complications

The results of uni- and multivariate analysis of postoperative complications are shown in Table 3. On multivariate analysis ≥75 years of age (OR, 2.08; 95% CI, 1.14–3.80; *p* = 0.016) and preoperative bowel obstruction (OR, 2.11; 95% CI, 1.20–3.70; *p* = 0.010) were risk factor for postoperative complications after emergency groin hernia repair.

**TABLE 3:** Univariable and Multivariable Analysis of complications, mortality and recurrence.

Variables	Univariable analysis						Multivariable analysis					
	Complications		Mortality 90 days		Recurrence		Complications		Mortality 90 days		Recurrence	
	OR (95%CI)	*p* value	OR (95%CI)	*p* value	HR (95%CI)	*p* Value	OR (95%CI)	*p* Value	OR (95%CI)	*p* Value	HR (95%CI)	*p* Value
Patient age (y)												
<75 (*n* = 125)	1	<0.001	1	<0.001	1	0.884	1	0.016	1	0.288		
≥75 (*n* = 191)	3.82 (2.36–6.20)		9.26 (2.15–39.84)		1.06 (0.49–2.27)		2.08 (1.14–3.80)		3.17 (0.38–26.61)			
Gender												
Male (*n* = 147)	1	0.948	1	0.003	1	0.005			1	0.055	1	0.011
Female (*n* = 169)	0.99 (0.63–1.53)		0.27 (0.11–0.67)		0.32 (0.14–0.71)				0.21 (0.04–1.03)		0.35 (0.15–0.78)	
BMI												
<30 (*n* = 250)	1	0.445	1	0.714	1	0.519						
≥30 (*n* = 31)	0.75 (0.35–1.59)		1.38 (0.38–4.98)		0.62 (0.15–2.63)							
ASA score												
I/II (*n* = 179)	1	<0.001	1	<0.001	1	0.955	1	0.203	1	0.518		
III/IV (*n* = 137)	2.89 (1.82–4.58)		4.20 (1.72–10.25)		1.02 (0.46–2.25)		1.46 (0.82–2.61)		0.63 (0.16–2.54)			
Charlson score												
<3 (*n* = 28)	1	<0.001	1	0.149	1		1	0.393				
≥3 (*n* = 288)	6.34 (2.15–18.74)		∞ (0.86 - ∞)		2.86 (0.39–21.14)		1.74 (0.49–6.18)					
Previous abdominal surgery												
Yes (*n* = 137)	0.82 (0.52–1.28)	0.376	0.89 (0.40–1.98)	0.774	0.79 (0.36–1.73)	0.558						
No (*n* = 179)	1		1		1							
Comorbidity												
Yes (*n* = 250)	3.50 (1.83–6.71)	<0.001	∞ (2.02–∞)	0.007	1.92 (0.66–5.59)	0.23						
No (*n* = 116)	1		1		1							
Cardiovascular disease												
Yes (*n* = 223)	2.71 (1.63–4.53)	<0.001	5.74 (1.33–24.77)	0.009	2.08 (0.83–5.17)	0.117	1.59 (0.80–3.15)	0.188	3.38 (0.33 –34.71)	0.306		
No (*n* = 93)	1		1		1		1		1			
COPD												
Yes (*n* = 65)	1.34 (0.77–2.31)	0.298	2.50 (1.09–5.77)	0.027	1.75 (0.74–4.14)	0.205			2.97 (0.7–11.96)	0.125		
No (*n* = 251)	1		1		1				1			
Chronic nephropathy												
Yes (*n* = 37)	2.87 (1.36–6.04)	0.004	5.71 (2.38–13.70)	<0.001	0.67 (0.09–4.98)	0.698	2.16 (0.90–5.18)	0.083	3.58 (0.82–15.66)	0.090		
No (*n* = 279)	1		1		1		1		1			
Liver cirrhosis												
Yes (*n* = 10)	1.64 (0.45–5.94)	0.444	1.0 (0.00–3.80)	1.000	3.67 (1.1–12.25)	0.034					2.39 (0.68–8.36)	0.173
No (*n* = 306)	1		1		1						1	
Diabetes												
Yes (*n* = 38)	1.23 (0.62–2.42)	0.551	0.91 (0.26–3.17)	1.000	0.51 (0.12–2.14)	0.354						
No (*n* = 278)	1		1		1							
Comorbidity more than one												
Yes (*n* = 167)	2.02 (1.29 – 3–17)	0.002	3.43 (1.34–8.74)	0.007	2.07 (0.95–4.51)	0.069					1.51 (0.67–3.41)	0.323
No (*n* = 149)	1		1		1						1	
Femoral hernia												
Yes (*n* = 179)	0.94 (0.61–1.47)	0.802	0.50 (0.22–1.10)	0.081	1.82 (0.77–4.31)	0.175			1.10 (0.21 – 5–76)	0.906		
No (*n* = 137)	1		1		1				1			
Recurrent hernia												
Yes (*n* = 56)	0.84 (0.47–1.51)	0.568	0.79 (0.26–2.39)	0.780	1.67 (0.71–3.96)	0.242						
No (*n* = 260)	1		1		1							
Necrotic contents												
Yes (*n* = 81)	4.83 (2.73–8.53)	<0.001	5.98 (2.61–13.69)	<0.001	1.90 (0.85–4.24)	0.118	2.75 (0.85–8.92)	0.093	7.04 (0.64–77.34)	0.111		
No (*n* = 235)	1		1		1		1		1			
Preoperative bowel obstruction												
Yes (*n* = 165)	4.61 (2.86–7.41)	<0.001	28.06 (3.76–209.53)	<0.001	0.64 (0.29–1.39)	0.260	2.11 (1.20–3.70)	0.010	8.00 (0.65–98.56)	0.105		
No (*n* = 151)	1		1		1		1		1			
Duration of incarceration												
<24 h (*n* = 124)	1	0.046	1	0.132	1	0.105	1	0.518				
≥24 h (*n* = 190)	1.59 (1.01–2.51)		1.97 (0.81–4.80)		0.53 (0.24–1.14)		1.19 (0.70–2.04)					
Bowel resection performed												
Yes (*n* = 66)	6.98 (3.55–13.71)	<0.001	4.91 (2.18–11.06)	<0.001	1.37 (0.55–3.39)	0.502	1.79 (0.48–6.74)	0.388	0.32 (0.03–3.14)	0.325		
No (*n* = 250)	1		1		1		1		1			
CCI												
<26.2 (*n* = 37)	1	<0.001	1	<0.001	1	0.947	NA		44.76 (4.51–444.59)	0.001		
≥26.2 (*n* = 46)	0.74 (0.30–1.85)		81.25 (10.74–614.68)		1.04 (0.37–2.88)				1			
Mesh repair						0.882				0.141		
Yes (*n* = 296)	1.0 (0.63–1.60)	0.523	0.24 (0.08–0.72)	0.020	1.16 (0.16–8.58)				0.25 (0.04–1.58)			
No (*n* = 20)	1		1		1				1			
Type of procedure												
Anterior (*n* = 206)	1	0.983	1	0.800	1	0.083					1	0.107
Preperitoneal open (*n* = 110)	4.83 (2.73–8.53)		1.11 (0.49–2.52)		0.39 (0.13–1.13)						0.42 (0.14–1.21)	
Type of mesh repair												
Lichtenstein (*n* = 61)	1		1		1							
Plug and patch (*n* = 21)	1.22 (0.45–3.31)	0.691	1.50 (0.25–8.85)	0.654	0.74 (0.08–6.67)	0.792						
Mesh plug (*n* = 107)	1.32 (0.70–2.49)	0.389	1.00 (0.28–3.56)	0.997	2.08 (0.7–6.2)	0.188						
Preperitoneal mesh (*n* = 107)	1.27 (0.68–2.40)	0.456	1.31 (0.39–4.44)	0.666	0.63 (0.16–2.51)	0.510						
Intraoperative complications												
Yes (*n* = 17)	5.44 (1.53–19.34)	0.004	5.25 (1.69 –16.24)	0.009	0.36 (0.02–6.32)	0.486	4.08 (0.99–16.91)	0.052	1.11 (0.19–6.50)	0.905		
No (*n* = 299)	1		1		1		1		1			

### Risk Factors of 90-day Mortality

Table 3 shows the results of uni- and multivariate analysis of 90-day mortality. Multivariate analysis identified CCI ≥26.2 (OR, 44.76; 95% CI, 4.51–444.59; *p* = 0.01) as a risk factor for 90-day mortality after emergency groin hernia repair.

### Risk Factors of Recurrence

Table 3 shows the results of uni- and multivariate analysis of recurrence. In the multivariate analysis, only the female gender (HR, 0.35; 95% CI, 0.15–0.78; *p* = 0.011) was a risk factor for recurrence after emergency groin hernia repair.

## Discussion

In the present study significant advantages were identified in the open preperitoneal repair over anterior approach in terms of less need for associated midline laparotomies and lower recurrence rate. In patients in whom potential intestinal resection was more expected (femoral hernia or longer duration of incarceration) the more frequent used techniques were open preperitoneal, mesh plug and tissue repair. Age ≥75 years and preoperative intestinal obstruction were independent factors associated with postoperative complications. CCI ≥26.2 was significantly associated with increased mortality at 90 days and female gender was the factor correlated with hernia recurrence.

Regarding the short-term outcomes, in our series there were no differences between the groups according to surgical approach in terms of postoperative complications or length hospital stay. However, in the comparison by type of technique repair, we observed that tissue repair presented higher CCI^®^ compared to mesh repairs. This higher severity of postoperative complications could be related to the high number of contaminated surgeries, higher frequency of necrotic hernia content and intestinal resections present in the tissue repair group. These findings are consistent with previously published literature and confirm that mesh repairs are safe in the emergency setting [2,3]. On the other hand, in our study preperitoneal mesh repair was associated with fewer midline laparotomies, even though this group had a higher proportion of bowel resections. Similar results were reported by others [5]. In our series the patients were operated on using an extensive preperitoneal approach [7]. Through this extensive approach, it was possible to have access to the peritoneal cavity for the inspection of the herniated content, allowing for comfortable intestinal resections if needed, also being able to have a complete view of the myopectineal orifice and assess other potential hernias, as well as placing a mesh covering the entire area. In our opinion this is an important finding since midline laparotomy in emergency groin hernia repair can reach up to 53.1% [17] and it has been identified as a prognostic factor for postoperative morbidity [18]. However, our data seems rather to suggest that the need for an additional midline laparotomy and the decision to perform a non-mesh repair were not influenced by the initial approach as open preperitoneal or anterior.

The open preperitoneal method also was associated with significantly lower rates of recurrence, both by type of approach and by techniques. Recurrence rates after emergency groin hernia repair range from 0.9% to 10% [3,4,8,19,20]. In our study it was 8.5% (*n* = 27) and in the majority of cases was after a mesh plug repair (*n* = 17). In light of these results and following current clinical guidelines, the mesh plug repair should be avoided [1]. On the other hand, multivariate analysis indicated that female gender was the only risk factor for recurrence after emergency groin hernia repair, which is consistent with previous data [21]. A hypothesis for the higher recurrence rate in females could be that femoral hernias were missed at the primary procedure [22]. These findings make the open preperitoneal technique very attractive in the emergency setting, since it allows a complete exposure of the myopectineal orifice, being able to identify all possible hernias in the inguinofemoral region. The open preperitoneal mesh repair technique used in this study consists of creating a gap in the mesh for the passage of the inguinal cord elements. However, it is still unknown whether making a gap in the mesh would lead to a higher rate of recurrence or chronic pain.

The incidence of chronic postoperative inguinal pain in the present study was 2.2% without significant differences according to the type of approach and repair technique, while rates of 0.7%–75% have been reported in open hernia mesh repairs depending on the definitions of chronic postoperative inguinal pain and assessments methods [23]. In the context of emergency repairs, a rate of 5% has been reported [20]. A possible explanation for this relatively low incidence of chronic pain may be the high number of elderly patients and that the frequency of chronic postoperative inguinal pain decreases with age [24].

Different open surgical techniques have been described where the purpose is to place the mesh into the preperitoneal space [25]. However, a limited number of studies have reported the results of using the open preperitoneal approach in emergency groin hernia repair. Pans et al published one of the first studies describing 35 patients treated by insertion of a prosthetic mesh via midline preperitoneal approach. They concluded that the preperitoneal prosthesis implantation is safe, even when necrotic intestine or omentum was resected [7]. Karatepe et al reported the only randomized study comparing open posterior vs. open anterior approach with mesh, found no significant differences except for a lower incidence of second incisions in the posterior approach [6]. In a recent retrospective study, the authors included 146 patients and reported a total of 15 patients (10.3%) who developed complications, no mesh were removed, and 2 patients had recurrence with a median follow-up of 26 months [8]. Regarding the use of laparoscopic approach in emergency groin hernia repair, some authors have reported good results in selected patients, especially with TAPP approach [5]. However, some drawbacks have been described that have prevented a more widespread use of this approach in this context. Among the difficulties for the implementation of laparoscopy is the bowel distention that is frequently observed in these patients and can lead to conversion to open surgery and visceral injuries derived from laparoscopic manipulation [26]. Unlike the laparoscopic posterior approach, the open posterior approach is not limited to selected patients. With the open posterior approach, the possibility of visceral injury from manipulation is reduced and the presence of bowel distention is not an inconvenience for its performance. Therefore, our experience confirms that open preperitoneal repair using a posterior approach is effective and safe in the difficult setting of incarcerated/strangulated groin hernia.

In our study the morbidity rate was 48.1%, with 14.8% of major complications and a mortality of 8.5%, these numbers are substantially higher than those reported in other similar studies [2,4,8,17,18,19]. The explanation for these findings may be influenced by the fact that in our series a significant number of patients were elderly with high comorbidity. This is reflected in the fact that 60% of the patients were older than 75 years, with a high comorbidity represented by the fact that 43.4% were ASA II/IV. On the other hand, 28% of the patients underwent intestinal resection. These factors have been significantly associated with morbidity and mortality after emergency repair of abdominal wall hernias [27]. In line with the foregoing, in our multivariate analysis, patients older than 75 years and preoperative bowel obstruction were independent risk factors for postoperative morbidity, as described in previous studies [28]. On the other hand, CCI^®^ was the only independent risk factor for mortality at 90 days in our series. It has recently been shown that CCI^®^ can be a more accurate scale for measuring morbidity in high-risk patients with the probability of multiple complications [29]. To our knowledge, this is the first emergency groin hernia repair study to report postoperative morbidity using this risk scale. According to these findings, elderly patients with associated comorbidities, and especially women, could benefit from elective inguinal hernia repair to avoid the risks of emergency intervention for inguinal hernia, as reported in previous studies [4,30].

This study has several limitations: 1. observational study of a single center experience; 2. inconsistency in follow-up schedule in terms of limited number of patients followed up; 3. despite exhaustive efforts, not all the patients could be contacted by telephone for follow-up, so the reported recurrence and postoperative chronic inguinal pain rates could potentially underestimate the current rate. All would lead to inevitable bias and potentially underestimating hernia recurrence and long-term complication rates. Despite these limitations, our study provides new evidence on the clinical comparison of surgical approach in emergency groin hernia repair with a high number of patients.

In conclusion, this study has shown that the open preperitoneal approach was associated with lower rates of recurrence and associated midline laparotomy. Open preperitoneal access may be a good choice in the of context intestinal resection to avoid the morbidities associated with additional midline laparotomies. Mesh plug had a higher recurrence rate. The rest of anterior approaches were safe and effective in emergency groin hernia repair, and this can justify the choice of approach at the surgeon´s discretion.

## Data Availability

The raw data supporting the conclusion of this article will be made available by the authors, without undue reservation.
